# Bloqueio do Ramo Esquerdo Idiopático e Sintomas Inexplicáveis Durante o Exercício: Um Relato de Caso

**DOI:** 10.36660/abc.20190363

**Published:** 2020-09-11

**Authors:** Guilherme Veiga Guimarães, Edimar Alcides Bocchi

**Affiliations:** 1 Universidade de São Paulo Instituto do Coração São PauloSP Brasil Universidade de São Paulo Instituto do Coração,São Paulo, SP – Brasil

**Keywords:** Bloqueio de Ramo, Exercício, Atividade Física, Consumo de Oxigênio/fisiologia, Doenças Cardiovasculares/prevenção e controle

## Introdução

A presença de bloqueio do ramo esquerdo (BRE) na aparente ausência de outra doença cardíaca suscita questões e preocupações a respeito da estratificação de risco de eventos ou sintomas cardiovasculares subsequentes.^1 - 5^ Estima-se que a detecção de BRE em adultos assintomáticos, inclusive em atletas, varie entre 0,1% e 0,8%, o que muito provavelmente corresponde a doenças cardíacas estruturais, e não a respostas fisiológicas ao exercício.^6 - 8^ Por outro lado, alguns estudos demonstraram que o risco de morte dos pacientes com BRE e doença cardíaca varia entre 2,4% e 11% ao ano.^9^

Embora vários estudos tenham sugerido que o BRE induzido por exercício esteja geralmente associado à doença cardiovascular, sobretudo à doença arterial coronariana, há estudos contrastantes que demonstram uma relação entre o BRE induzido por exercício e artérias coronárias normais.^6 , 7 , 9^ Entretanto, os efeitos cardiovasculares adversos relacionados ao exercício no BRE com função cardíaca normal em repouso ainda são pouco definidos.

Este relato de caso examinou a relação entre o exercício, o BRE, sintomas e capacidade de exercício em mulher mais jovem com BRE típico, sem história de doença cardiovascular, que relatou ansiedade e falta de ar súbitas durante exercício vigoroso, o que pode ser sugestivo de doença cardíaca, tendo sido encaminhada para teste de esforço.

## Relato de Caso

Mulher saudável de 42 anos de idade com BRE, que relatou ansiedade e falta de ar súbitas durante exercício vigoroso, encaminhada para teste cardiopulmonar do exercício (TCPE) para avaliação dos sintomas inexplicáveis. Ela não estava tomando nenhuma medicação e não tinha histórico médico relevante. Sem sintomas prévios sugestivos de doença cardíaca (desconforto no peito, palpitações, desmaios e angina). Não havia história de doença neuromuscular ou pulmonar. Ela não fumava nem consumia álcool. Não havia história familiar de doença cardíaca ou ataque cardíaco. Nos seis meses anteriores, ela vinha se exercitando três vezes por semana em uma academia. O programa de exercício consistia em sessões de pelo menos 60 minutos de atividade regular de intensidade moderada, incluindo exercícios aeróbicos, de fortalecimento e flexibilidade muscular e de fortalecimento do equilíbrio. Seu exame físico foi considerado normal, o IMC era 21,5 kg/m^2^ e a pressão arterial em repouso estava em 110/70 mmHg. O eletrocardiograma (ECG) em repouso mostrou ritmo sinusal (RS) e frequência cardíaca (FC) de 70 bpm, com característica dominante de bloqueio intraventricular: complexo QRS prolongado (≥0,12s) resultante do atraso na ativação do ventrículo esquerdo, acompanhado de morfologia característica do complexo QRS.6 A angiotomografia coronariana (ATC) foi realizada, e não mostrou depósitos de cálcio e gordura nas artérias coronárias, nem artérias coronárias com estenose. O hemograma completo mostrou resultados normais: glicemia de jejum: 78 mg/dL; colesterol lipoproteína de baixa densidade (LDL): 168 mg/dL; colesterol lipoproteína de alta densidade (HDL): 81 mg/dL; colesterol total (CT): 159 mg/dL; lipoproteína (a) [Lp(a)]: 7 mg/dL; triglicérides (Tg): 49 mg/dL e creatina fosfoquinase (CPK): 26 U/L. A ressonância magnética (RM) do coração mostrou função biventricular normal, fração de ejeção do VE de 65% e dimensões preservadas, exceto por um movimento septal anormal.

**Figura 1:**
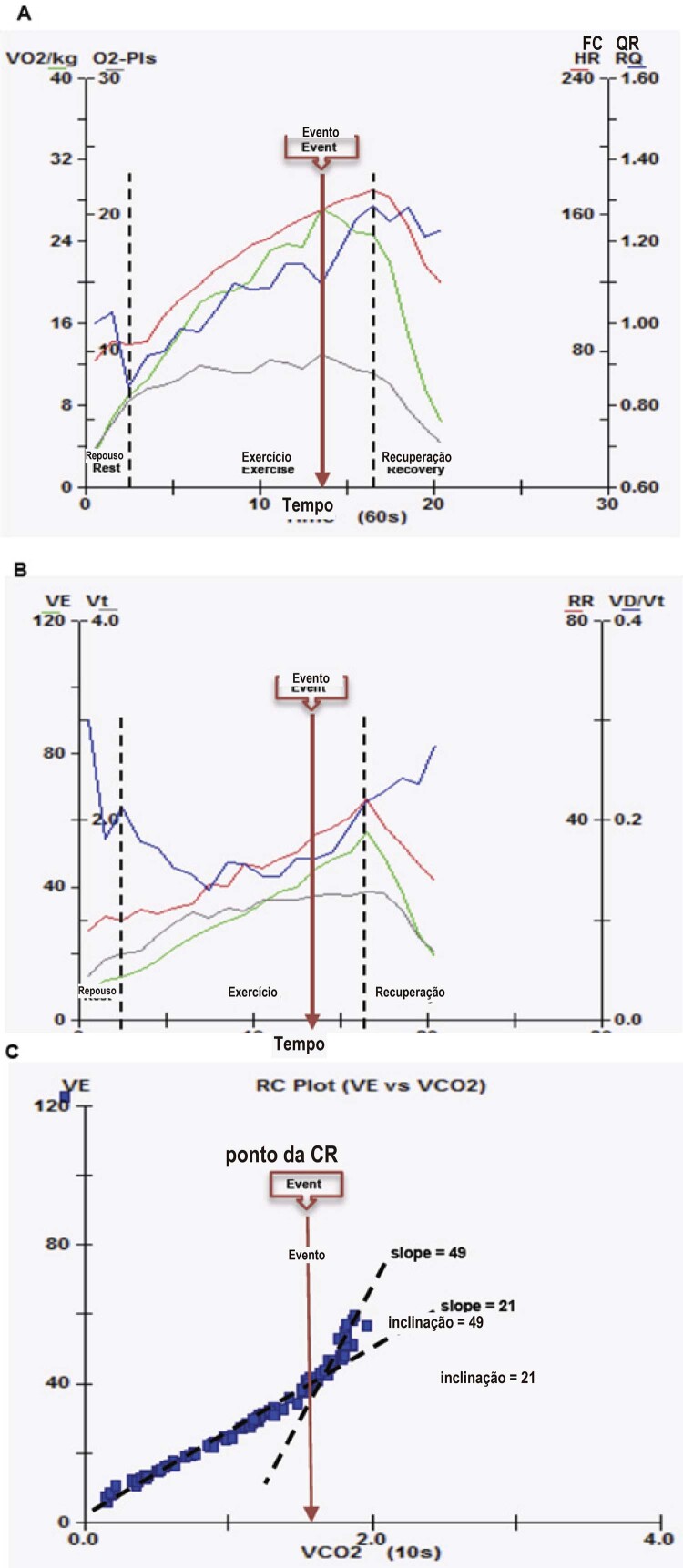
– Teste cardiopulmonar do exercício em repouso, durante o exercício e a recuperação. As linhas pretas indicam a transição entre essas fases. A) linha verde – consumo de oxigênio (VO2/kg, ml/kg/min), linha vermelha – frequência cardíaca (FC, bpm), linha preta – pulso de oxigênio (O2-Pls, ml/bpm) e linha azul – quociente respiratório (QR); B) linha verde – ventilação por minuto (VE, l/min), linha vermelha – taxa respiratória (RR), linha preta – volume corrente (Vt, ml/min) e linha azul - razão espaço morto/volume corrente (Vd/Vt). C) Inclinação da relação entre ventilação minuto/produção de dióxido de carbono (VE/VCO2). Na primeira parte do exercício, a inclinação VE/VCO2 está normal (21); a partir do momento do evento, durante o teste de esforço, a inclinação VE/VCO2 aumentou vertiginosamente (49). Seta marrom – momento do evento.

**Figura 2:**
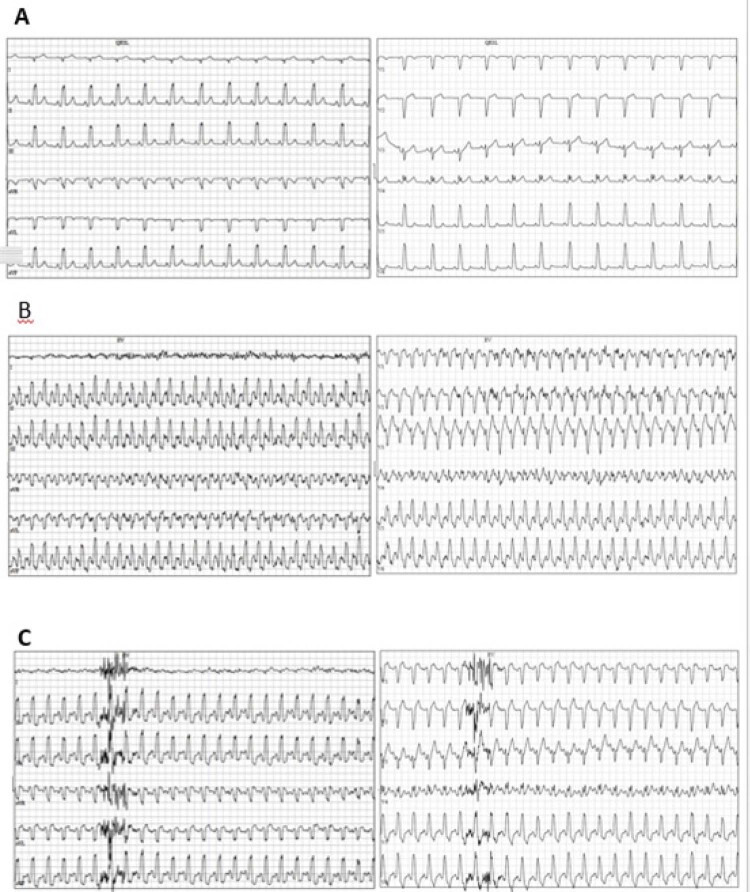
– Eletrocardiograma demonstrando bloqueio de ramo esquerdo: taxa de repouso de 75 bpm (A), frequência cardíaca de 175 bpm durante o teste de tolerância ao exercício máximo (B) e frequência cardíaca de 153 bpm no primeiro minuto do período de recuperação (C).

Foi submetida ao TCPE em uma esteira. Ao longo do TCPE, o ECG de 12 derivações mostrou ritmo sinusal ( Figura 1 ). As aferições da pressão arterial estavam dentro da normalidade: em repouso (126/82 mmHg), pico (160/90 mmHg) e recuperação (120/90 mmHg). Ela parou o exercício por causa da fadiga (RER=1,29). Os valores de pico de consumo de oxigênio (VO_2_, pico = 27,1 ml/kg/min) e frequência cardíaca máxima (FCM = 176 bpm) obtidos no TCPE foram normais para a idade e o sexo: 95% e 102%, respectivamente (https://www.ahajournals.org/doi/10.1161/01.CIR.91.2.580). Do estágio 14 do protocolo de Balke modificado até o final do teste, o TCPE identificou uma redução do VO_2_ e do pulso O_2_ (VO_2_/FC, ml/bpm), e um aumento da FC e da relação espaço morto/volume corrente (Vd/Vt) ( Figura 2 ). A partir deste evento, a inclinação da relação entre ventilação minuto/produção de dióxido de carbono (VE/VCO_2_) aumentou abruptamente e não foi acompanhada de hipóxia ( Figura 2 ).

## Discussão

Tanto quanto é do nosso conhecimento, relatamos pela primeira vez o caso de uma paciente mais jovem com BRE sem cardiomiopatia aparente e com sintomas inexplicáveis durante exercício vigoroso (redução no VO_2_ durante o TCPE), o que é sugestivo de função cardíaca comprometida como consequência de estresse cardiovascular. O ECG não revelou anomalias, a não ser pelo BRE. A reserva de frequência cardíaca e a pressão arterial permaneceram normais ao longo do TCPE.

O exercício oferece uma ferramenta útil para a avaliar indiretamente a reserva funcional cardíaca, a partir do desempenho ventricular esquerdo, as alterações do repouso para o pico do esforço, podendo ser limitado na presença de doenças. Nesse contexto, uma queda no padrão cardiovascular de resposta do VO_2_ pode indicar um comprometimento do débito cardíaco (DC) ou da extração periférica de oxigênio, conforme observado na insuficiência cardíaca.10 Por outro lado, nos indivíduos saudáveis, o nível de aumento do consumo e extração periférica de oxigênio em resposta ao exercício é muito maior, quando comparado com as alterações no volume sistólico, e semelhante ao aumento observado na FC.

A redução do VO_2_ e do pulso O_2_, apesar do aumento na FC, observada nesse caso, pode indicar uma possível anomalia cardíaca. Sugerimos que o movimento assíncrono do ventrículo esquerdo, com o atraso da contração de suas paredes, pode diminuir a carga de trabalho do ventrículo esquerdo, resultando em um volume sistólico mais baixo e indicando uma diminuição do débito cardíaco durante o exercício máximo, apesar do aumento na FC.^8 - 10^ Essa redução na carga de trabalho do ventrículo esquerdo por um aparente defeito de perfusão septal durante o TCPE pode ocasionar perda de energia e “desperdício” de trabalho miocárdico, o que pode representar o impacto hemodinâmico da ativação elétrica assíncrona do miocárdio no BRE,^8 - 10^ além de explicar, em parte, a queda do VO_2_ observada durante o TCPE. Por outro lado, o bloqueio do ramo esquerdo induzido por exercício pode estar relacionado ou não a alterações cardíacas aparentes. Entretanto, os pacientes com este achado apresentaram taxas de mortalidade por todas as causas significativamente maiores se comparados àqueles sem bloqueio do ramo esquerdo induzido por exercício.^11^

Essa aparente diminuição da função cardíaca durante o exercício pode ser decorrente de uma diminuição transitória do volume sistólico, provavelmente relacionada a uma piora da função ventricular esquerda (VE), associada a um aumento repentino da inclinação VE/VCO_2_, dando origem a uma maior razão entre o espaço morto e o volume corrente (Vd/Vt) e a um aumento precoce da frequência respiratória, como mecanismos compensatórios.^1 , 3 , 5 , 10^ Esse distúrbio na fisiopatologia está relacionado com a disfunção do VE, provocada ou piorada pelo BRE, o que pode ocasionar indiretamente uma disfunção ventricular direita, por meio do aumento da pressão de enchimento do lado esquerdo, causando alterações na função das vias aéreas dos pulmões, e o surgimento de troca anormal de gases em decorrência da disfunção alvéolo-capilar.^3 , 4^ Além disso, uma inclinação VE/VCO_2_ mais elevada é indicativa de hipertensão pulmonar secundária, como consequência de outras condições primárias, tais como insuficiência cardíaca ou doença pulmonar.^3 , 4^

## Consideração

O teste cardiopulmonar do exercício no BRE, na ausência de outras doenças cardíacas, deve ser considerado como uma técnica para avaliar a capacidade de exercício em pacientes com sintomas inexplicáveis.
