# Supplementary material for: Bloqueio do Ramo Esquerdo Idiopático e Sintomas Inexplicáveis Durante o Exercício: Um Relato de Caso
Source: Arq Bras Cardiol. 2020 Sep 11;115(1 Suppl 1):10–3. [Article in Portuguese] doi: 10.36660/abc.20190363 (PMC8386955; doi:10.36660/abc.20190363)
Supplement: Translation

# Translation: Idiopathic Left-Bundle Branch Block and Unexplained Symptom At Exercise: A Case Report

**Authors:** Guilherme Veiga Guimarães, Edimar Alcides Bocchi

**Affiliations:** 1 Brasil Universidade de São Paulo Instituto do Coração, São Paulo, SP – Brasil

**Keywords:** Bundle Branch Block, Exercise, Physical Activity, Oxygen Consumption/physiology, Cardiovascular Diseases/prevention and control

## Introduction

The presence of a left bundle branch block (LBBB) in the apparent absence of any other heart disease raises questions and concerns about the stratification of the risk of subsequent cardiovascular events or symptoms.^1 - 5^ The detection of LBBB in asymptomatic adults, including athletes, is estimated to range between 0.1% and 0.8%, which is more likely to represent a structural heart disease rather than a physiological response to exercise.^6 - 8^ On the other hand, some studies have shown that the mortality risk of patients with LBBB and heart disease varies between 2.4% and 11% per year.^9^

Although several studies have suggested that exercise-induced LBBB is usually associated with cardiovascular disease and, particularly, coronary artery disease, there are contrasting studies showing an association between exercise-induced LBBB and normal coronary arteries.^6 , 7 , 9^ However, exercise-related cardiovascular adverse effects in LBBB with normal resting cardiac function remains poorly defined.

This case report examined the relationship between exercise, LBBB, symptoms and exercise capacity in a younger woman, with typical LBBB and without history of cardiovascular disease, who reported sudden anxiety and shortness of breath during vigorous exercise, which can be suggestive of cardiac disease, being referred for exercise stress testing.

## Case Report

A healthy 42-year-old woman with LBBB, who reported sudden anxiety and shortness of breath during vigorous exercise, and was referred for cardiopulmonary exercise testing (CPX) to evaluate the unexplained symptoms. She was not taking any medication and had no significant medical history. She had no previous symptoms suggestive of cardiac disease (chest discomfort, palpitations, fainting and angina). She had no history of neuromuscular or pulmonary disease. She did not smoke or drink alcohol. There was no family history of cardiac disease or heart attack. Over the previous 6 months, she had been exercising three times a week at the gym. The exercise program consisted of sessions of at least 60 min of regular activity at moderate intensity, including aerobic, muscle-strengthening, flexibility, and balance-strengthening exercises. Her physical examination was considered normal, her BMI was 21.5 kg/m^2^and her resting blood pressure was 110/70 mmHg. The resting electrocardiogram (ECG) showed a sinus rhythm (SR) and heart rate (HR) of 70 bpm, with the dominant feature of intraventricular block: prolonged QRS complex (≥0.12s) due to delayed activation of the left ventricle, accompanied by a characteristic morphology of the QRS complex.^6^ Coronary computed tomography angiography (CTA) was performed, which showed no deposits of calcium and fatty material in the coronary arteries and no stenotic coronary arteries. A complete blood count showed normal results: her fasting glucose was 78 mg/dL, low-density lipoprotein cholesterol (LDL-C) was 168 mg/dL, high-density lipoprotein cholesterol (HDL-C) was 81 mg/dL, total cholesterol was 159 mg/dL, lipoprotein(a) [Lp(a)] was 7 mg/dL, triglycerides were 49 mg/dL and creatine phosphokinase (CPK) was 26 U/L. The magnetic resonance imaging (MRI) of the heart showed normal biventricular function, a left ventricular ejection fraction of 65% and preserved dimensions, except for an abnormal septal motion.

She underwent CPX on a treadmill. Throughout the CPX phases, the 12-lead ECG showed a SR ( Figure 1 ). Blood pressure measurements were in the normal range: at rest (126/82 mmHg), peak exercise (160/90 mmHg) and recovery stages (120/90 mmHg). She stopped the exercise due to fatigue (RER=1.29). Peak oxygen consumption (VO_2peak_= 27.1 ml/kg/min) and maximum heart rate (HRmax = 176 bpm) values obtained from CPX were normal for age and gender, 95% and 102%, respectively. (https://www.ahajournals.org/doi/10.1161/01.CIR.91.2.580) From stage 14 of the modified Balke protocol until test termination, the CPX identified a decrease in VO_2_and in O_2_pulse (VO_2_/HR, ml/bpm), and an increase in HR and dead space to tidal volume ratio (Vd/Vt) ( Figure 2 ). From this event, the minute ventilation/carbon dioxide production relationship slope (VE/VCO_2_slope) increased abruptly and was not accompanied by hypoxia ( Figure 2 ).

**Figure 1:** – Cardiopulmonary exercise test at rest, during exercise and recovery. The black lines indicate the transition between these phases. A) green line - oxygen consumption (VO2/kg, ml/kg/min), red line - heart rate (HR, bpm), black line - oxygen pulse (O2-Pls, ml/bpm) and blue line - respiratory coefficient (RQ); B) green line - minute ventilation (VE, l/min), red line - respiratory rate (RR, bpm), black line - tidal volume (Vt, ml/min) and blue line - dead space to tidal volume ratio (Vd/Vt). C) Minute ventilation/carbon dioxide production relationship slope (VE/VCO2 slope). In the first part of the exercise, VE/VCO2 slope is normal (21); from the event point during the exercise test, the VE/VCO2 slope severely increased (49). Brown line arrow - event point.

**Figure 2:** – Electrocardiogram demonstrating left bundle branch block: a resting rate of 75 bpm (A), a heart rate of 175 bpm during maximal exercise tolerance test (B) and a heart rate 153 bpm in the first minute of the recovery period (C).

## Discussion

To the best of our knowledge, we report for the first time a younger woman with LBBB without apparent cardiomyopathy and with unexplained symptoms during vigorous exercise (decreased VO_2_, during CPX), which is suggestive of impaired cardiac function in the face of cardiovascular stress. There were no ECG abnormalities except for a LBBB. Heart rate reserve and blood pressure were normal throughout the CPX test.

Exercise provides a useful tool for the indirect assessment of cardiac functional reserve, from left ventricular performance, the change from rest to peak exercise, and can be limited in the presence of disease. In this context, a drop in the cardiovascular pattern of VO_2_responses may represent impaired cardiac output (CO) or impaired peripheral oxygen extraction as is observed in heart failure.^10^ On the other hand, in healthy individuals, the level to which oxygen consumption and peripheral oxygen extraction increase in response to exercise is much greater compared to changes in stroke volume and similar to the increase observed in HR.

The decrease in VO_2_and O_2_pulse, in spite of the increase in HR, observed in this case, may indicate a possible cardiac abnormality. We suggest that the asynchronous motion of the left ventricle associated with delayed wall contraction can reduce the left ventricular workload, resulting in a lower stroke volume and indicating a decrease in cardiac output during maximal exercise, despite the increase in HR.^8 - 10^ This reduction in left ventricular work by an apparent septal perfusion defect during CPX can lead to energy loss and waste of myocardial work, which may represent the hemodynamic impact of asynchronous electrical activation of the myocardium during LBBB,^8 - 10^ and partly explain the drop of VO_2_observed during CPX. On the other hand, exercise-induced left bundle branch block may be related or not to apparent cardiac abnormalities. However, patients with this finding have significantly higher all-cause mortality rates compared to those without exercise-induced left bundle branch block.^11^

This apparent decrease in cardiac function during exercise may be due to a transitory decrease in stroke volume, probably related to worsening left ventricular (LV) function associated with an abrupt increase in the VE/VCO_2_slope, giving rise to a higher dead space to tidal volume ratio and an early increase in the respiratory rate, as compensatory mechanisms.^1 , 3 , 5 , 10^ This disturbance in pathophysiology is linked with LV dysfunction, caused or worsened by LBBB, may indirectly lead to right ventricular dysfunction through increased left-sided filling pressure, causing changes in the function of the lungs’ airways, and to the development of abnormal gas exchange due to alveolar-capillary dysfunction.^3 , 4^ In addition, a higher VE/VCO_2_slope is suggestive of secondary pulmonary hypertension, as a consequence of another primary condition, such as heart failure or pulmonary disease.^3 , 4^

## Consideration

Cardiopulmonary exercise testing in LBBB, in the absence of other heart diseases, should be considered as a technique to evaluate the exercise capacity in patients with unexplained symptoms.
